# Postoperative Staphylococcal Toxic Shock Syndrome in a Patient Following Right Knee Fracture Repair: A Case Report

**DOI:** 10.7759/cureus.93013

**Published:** 2025-09-23

**Authors:** Nicole Nyamongo, James Garritano, Daniel Rosen, Theodore Rosen

**Affiliations:** 1 School of Medicine, Baylor College of Medicine, Houston, USA; 2 Dermatology, Baylor College of Medicine, Houston, USA; 3 Pathology and Immunology, Baylor College of Medicine, Houston, USA

**Keywords:** bone and joint, internal medicine, orthopedic surgery, severe bacterial infection, toxic shock syndrome

## Abstract

Toxic shock syndrome (TSS) is a rare but life-threatening multisystem disorder most commonly caused by *Staphylococcus aureus*. It is characterized by the acute onset of high fever, hypotension, a diffuse macular rash, and multiorgan involvement. We report the case of a male in his late 30s who sustained a patellar fracture with associated retinacular injury following trauma and subsequently underwent open reduction and internal fixation. Several days after discharge, he developed fever, chills, hypotension, and a pruritic, erythematous rash. He was readmitted and initially evaluated by the orthopedic service, which did not recommend additional imaging or surgical intervention, as they did not believe the knee to be the source of infection. The dermatology service was later consulted for rash evaluation and raised the possibility of TSS based on the patient’s constellation of systemic symptoms with diffuse erythematous rash. The differential diagnosis included drug eruption, such as Stevens-Johnson syndrome and drug reaction with eosinophilia and systemic symptoms (DRESS), though biopsy revealed spongiotic dermatitis.

Given the patient’s worsening shock presentation despite appropriate antibiotic therapy, dermatology recommended urgent re-evaluation of the surgical site to identify and treat the potential infectious source. The orthopedic team performed repeat imaging and surgical exploration, which revealed a methicillin-resistant *S. aureus* (MRSA) infection in the knee joint. This case underscores the importance of having a high index of suspicion for TSS in patients with systemic symptoms and cutaneous eruptions following orthopedic procedures. Recognition of characteristic cutaneous findings in a toxic-appearing postoperative patient should prompt consideration of TSS, as early diagnosis and surgical source control are essential to reducing morbidity and mortality.

## Introduction

Toxic shock syndrome (TSS) is a severe, acute-onset multisystem illness typically caused by *Staphylococcus aureus* and *Streptococcus pyogenes* (group A *Streptococcus*) [1]. TSS presents with high fever, gastrointestinal symptoms, a diffuse macular erythroderma, hypotension, and rapid progression to multiorgan dysfunction.

In the early 1980s, a notable outbreak of Staphylococcal TSS occurred among young women utilizing highly absorbent tampons, an event now referred to as “menstrual TSS” [1]. Since then, the incidence of menstrual TSS has significantly declined, attributed to advancements in tampon manufacturing and usage practices. The incidence of TSS peaked in 1980 at 14 cases per 100,000 menstruating women annually but has since declined to approximately 1 case per 100,000 persons [1-3]. TSS can also manifest in individuals undergoing surgical interventions or nasal packing procedures. Of the non-menstrual TSS, postoperative cases represent only a small fraction and are rarely reported in the literature. A systematic review identified fewer than 100 published cases of postoperative TSS worldwide, typically arising within 10 days of surgery [4]. The syndrome can arise from infections involving *S. aureus* strains that produce the TSS toxin-1 [1]. This toxin functions as a “superantigen,” binding to major histocompatibility complex (MHC) class II molecules on antigen-presenting cells and T-cell receptors, thereby triggering the release of cytokines and chemokines [1].

A systematic approach to the identification and management of TSS is critical to preventing disease progression and potentially fatal sequelae. The seven Rs of managing and treating TSS - recognition, resuscitation, removal of the infectious source, rational selection of antibiotics, role of adjunctive therapies, review of clinical progress, and reduction of risk among close contacts - provide an evidence-based framework for effective intervention [5]. TSS should be suspected in post-operative patients presenting with the triad of fever, hypotension, and diffuse rash, particularly when accompanied by multiorgan involvement and negative blood cultures. While local signs of infection may be initially subtle or absent, as demonstrated in our case, where initial orthopedic evaluation was negative, the systemic toxin-mediated syndrome requires urgent recognition and source control.

## Case presentation

**Figure 1:**
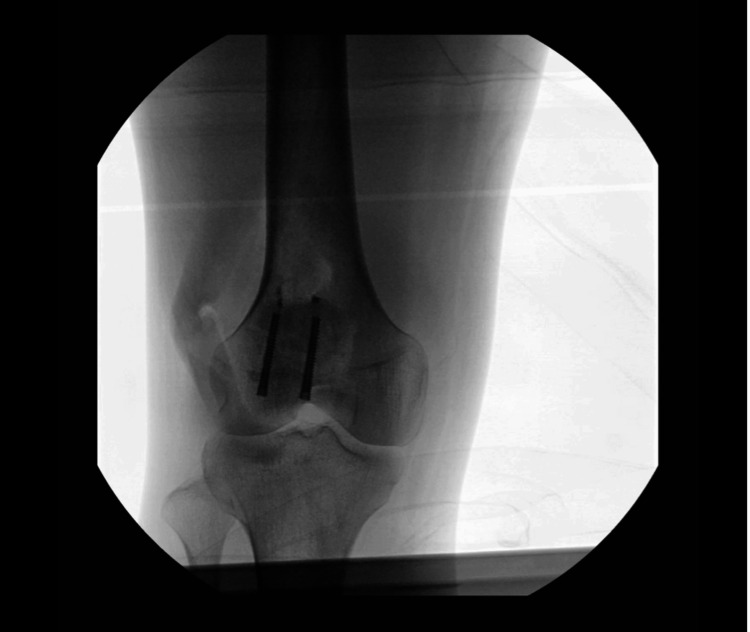
X-ray of the right knee after open reduction and internal fixation.

We report the case of a male in his late 30s who sustained a right knee injury following a fall. The imaging studies revealed a right patellar transverse fracture and medial and lateral extensor retinaculum tears. The patient underwent an open reduction and internal fixation procedure using Arthrex 5.0 cannulated screws (Arthrex, Naples, FL, USA) and received routine perioperative antibiotic prophylaxis with cephalexin (Figure 1). Following the surgery, he was discharged home with a continuous regional nerve block catheter placed in the right femoral region, which was removed four days later. The patient was prescribed tramadol, methocarbamol, and nonsteroidal anti-inflammatory drugs (NSAIDs) for subsequent pain management.

On post-operative day 3, the patient began experiencing fever (maximum temperature 101.6°F at home), chills, nausea, one episode of vomiting, dizziness, and increasing right knee pain. He also reported an erythematous, pruritic rash affecting the abdomen, back, and lower extremities. He presented to the emergency department on post-op day 6 with worsening symptoms and a systolic blood pressure of 74 mmHg. He received a two-liter intravenous fluid bolus, with improvement in vital signs, and did not require vasopressor support.

Laboratory findings were significant for leukocytosis (white blood cell count: 18 ×10^3^/µL), elevated lactic acid (6.4 mmol/L), acute kidney injury (creatinine: 2.0 mg/dL; baseline 0.93 mg/dL), procalcitonin (7.6 ng/mL), C-reactive protein (44.6 mg/L), erythrocyte sedimentation rate (75 mm/hr), and severe hyponatremia (sodium: 119 mEq/L) (Table 1). Additionally, his biochemical panel was consistent with hepatocellular injury. Empiric broad-spectrum antibiotics were initiated, including intravenous vancomycin and cefepime, and the patient was admitted for further workup.

**Table 1:** Laboratory findings on admission. AST: aspartate aminotransferase; ALT: alanine aminotransferase

Laboratory test	Patient values	Reference range
White blood cell (10^3^/μL)	18	3.5-10
Lactic acid (mmol/L)	6.4	0.5-2.2
Creatinine (mg/dL)	2.0	0.6-1.3
Procalcitonin (ng/mL)	7.6	≤0.09
C-reactive protein (mg/L)	44.6	0-0.7
Erythrocyte sedimentation rate (mm/hr)	75	0-0.15
Sodium (mEq/L)	119	136-145
AST (U/L)	115	10-40
ALT (U/L)	142	7-56
Total bilirubin (mg/dL)	7.4	0.2-1.0
Magnesium (mg/dL)	1.5	1.7-2.2
Platelet count (10^3^/μL)	175	150-450

**Figure 2:**
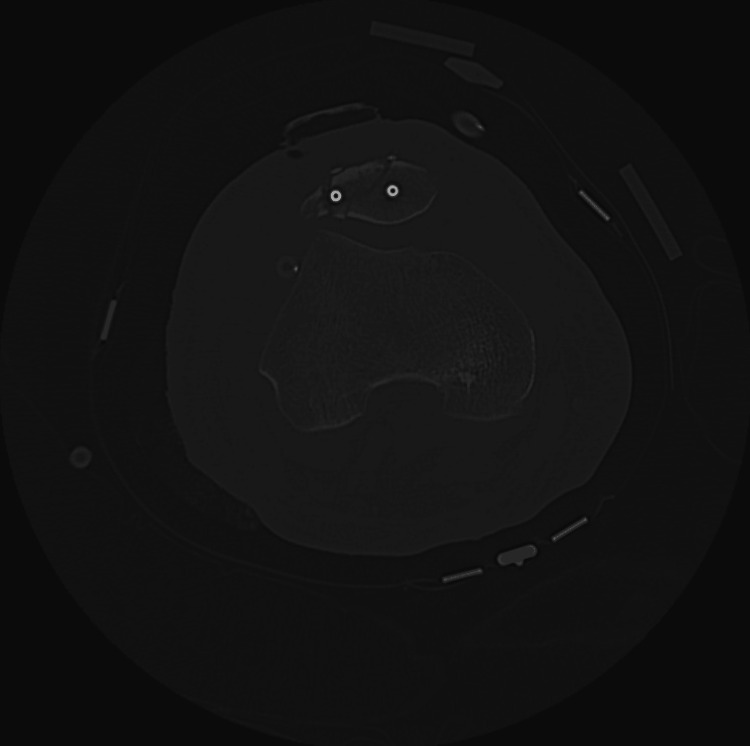
Computed tomography of the right knee showing nonspecific swelling and fluid collection in the anterior knee.

Computed tomography (CT) of the right knee showed postoperative changes with nonspecific anterior soft tissue swelling and fluid (Figure 2). Initial evaluation by the orthopedic service did not identify the knee as an infectious source.

**Figure 3:**
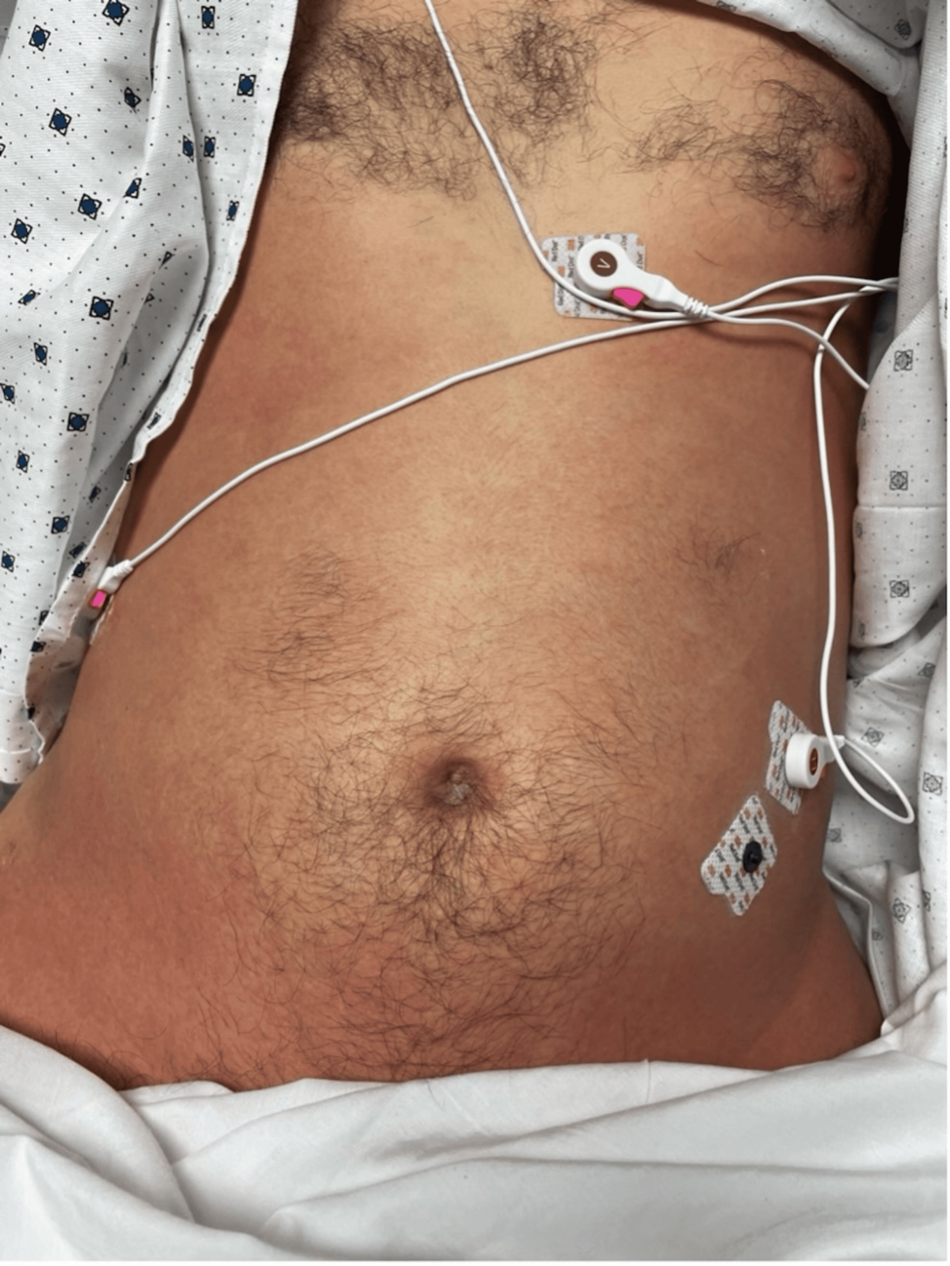
Uniform erythematous patch with indistinct borders on the patient’s abdomen.

**Figure 4:**
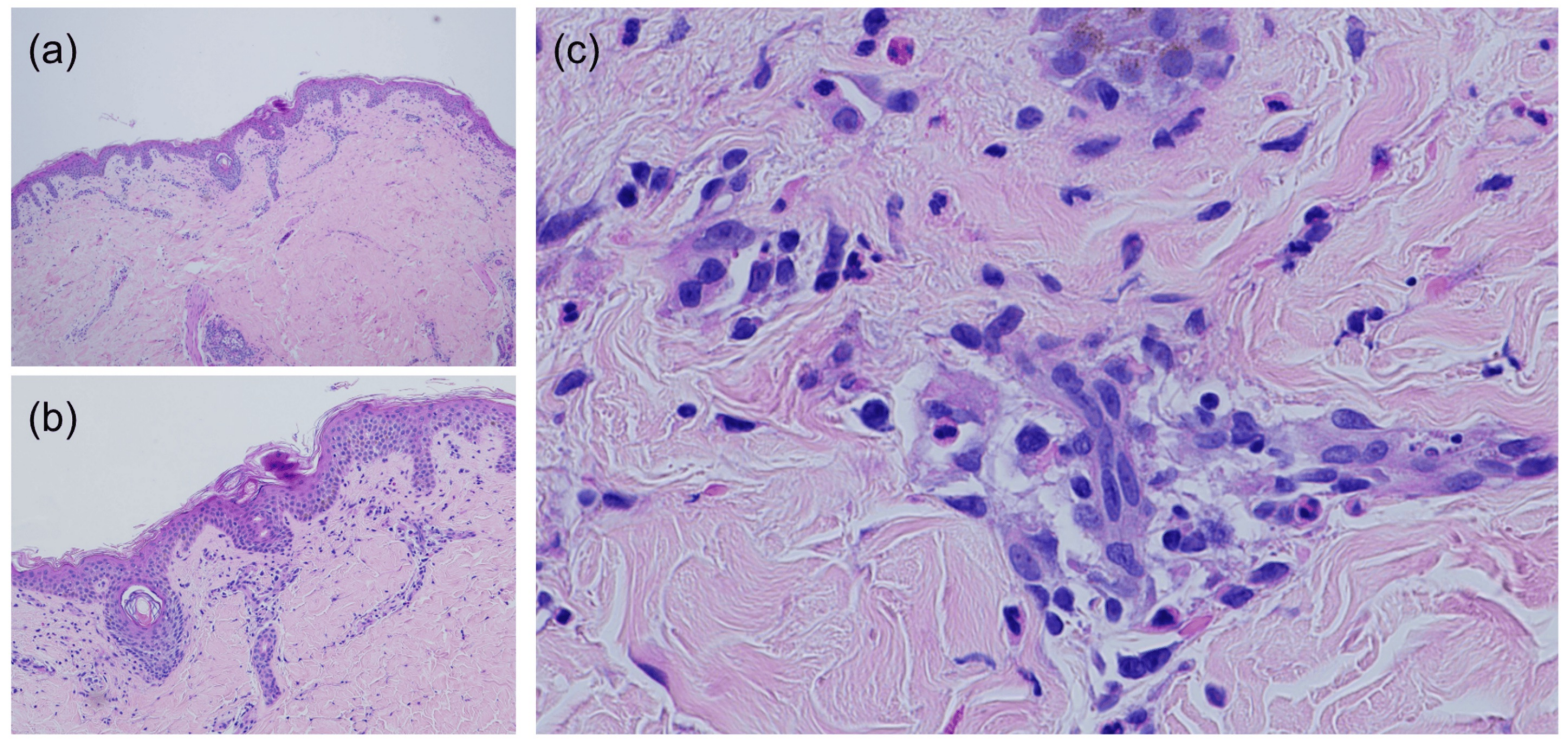
(a) Scant, mild superficial perivascular infiltrate; (b) scattered necrotic keratinocytes within the epidermis; (c) lymphocytes admixed with eosinophils in the superficial dermal inflammatory infiltrate.

On hospital day 2, the patient developed a generalized confluent erythematous rash, which prompted a dermatology consult to evaluate for possible drug eruption. The differential diagnoses included a morbilliform drug eruption (secondary to tramadol or perioperative cephalexin from the initial knee surgery), Stevens-Johnson syndrome, drug reaction with eosinophilia and systemic symptoms (DRESS), and TSS. A punch biopsy of the abdomen was performed, which showed spongiotic dermatitis but did not alter clinical management (Figures 3, 4a-4c). The combination of fever, hypotension, diffuse rash, and multiorgan involvement, as demonstrated by vomiting, abnormal liver function tests, and elevated creatinine, raised high suspicion for TSS, prompting the dermatology team to recommend urgent re-evaluation of the surgical site to identify a possible infectious source.

Within 24 hours, the right knee exhibited increased warmth, erythema, and bulla formation. Empiric antibiotic therapy was broadened to include doxycycline and clindamycin for toxin suppression. Incision and drainage by the orthopedic service revealed purulence within the joint and a superficial hematoma. The intraoperative cultures from the right knee joint grew methicillin-resistant *S. aureus* (MRSA), while blood cultures were negative. Antimicrobial susceptibility testing was performed, and the results for the isolated MRSA are shown in Table 2. The patient was treated with linezolid due to MRSA susceptibility and its anti-toxin properties. These findings confirmed the knee as the source of infection and, in conjunction with the patient’s systemic symptoms and cutaneous findings, established the diagnosis of TSS. The patient’s blood cultures remained negative throughout his hospitalization, which is consistent with the pathophysiology of TSS, where bacteremia is typically absent due to the toxin-mediated mechanism rather than direct bacterial invasion. TSS toxin 1 testing was not performed because the patient met the Centers for Disease Control and Prevention (CDC) clinical criteria for TSS, including the constellation of fever, hypotension, diffuse rash, multiorgan involvement, identification of a staphylococcal source, and negative blood cultures.

**Table 2:** Antimicrobial susceptibility testing. MIC: minimum inhibitory concentration; S: susceptible; R: resistant

Antibiotics	MIC (mg/L)	Interpretation
Cefazolin	≤8	R
Ceftriaxone	8	R
Ciprofloxacin	≤1	S
Clindamycin	0.5	S
Centamicin	≤4	S
Levofloxacin	≤1	S
Linezolid	2	S
Okacillin	>2	R
Penicillin	>2	R
Rifampin	≤1	S
Tetracycline	≤4	S
Trimethoprim-sulfamethoxazole	≤0.5/9.5	S
Vancomycin	1	S

Despite treatment, the patient remained systemically ill and underwent repeat right knee arthrotomy with irrigation and debridement. The surgical team placed a wound vacuum-assisted closure device to reduce local swelling and promote continuous drainage, preventing the risk of re-infection. The plastic surgery service performed additional debridement and noted extensive fat necrosis and soft tissue loss, which they later addressed by completing a gastrocnemius flap and skin graft for wound coverage.

Differential diagnosis

The primary admission diagnosis was septic shock, but was broadened to include morbilliform drug eruption, Stevens-Johnson syndrome, DRESS, and TSS after evaluation by dermatology. Drug-induced etiologies were considered due to exposure to tramadol and cephalexin, but were less likely given the absence of mucosal involvement, eosinophilia, periorbital edema, and the continued progression despite discontinuation of the medications. Autoimmune conditions and vasculitis were also considered but were excluded due to negative serologies and clinical context. Dermatology's concern for TSS, based on the CDC’s criteria for TSS, prompted the re-evaluation of the surgical site, which proved critical for definitive diagnosis.

Outcome and follow-up

Therapeutic linezolid was administered for six weeks and later transitioned to daptomycin prior to discharge, with an additional six weeks of treatment. Following discharge, the patient was placed on oral minocycline for chronic suppression therapy and a five-day course of oxycodone for pain control. He attended follow-up appointments with plastic and general surgery at one, two, four, and five weeks post-discharge. He received physical therapy and home wound care, reporting improvements in ambulation and pain.

## Discussion

The pathophysiology of TSS involves superantigens binding simultaneously to MHC class II molecules and T-cell receptors, bypassing normal antigen processing and leading to massive, uncontrolled cytokine release [1]. This mechanism explains the rapid progression from localized infection to systemic shock that can occur within hours. Diagnostically, TSS requires a high index of suspicion because early presentation may mimic other conditions such as viral syndromes, drug reactions, or sepsis. The key differentiating factors include the characteristic diffuse macular rash, the rapid progression to shock despite relatively localized infectious sources, and multiorgan involvement in the absence of bacteremia. Understanding these broader diagnostic principles is essential for clinicians across specialties, as delayed recognition significantly increases the risk of morbidity and mortality.

The first reported case of TSS following orthopedic procedures was in 1984, which involved an 18-year-old male who underwent elective joint fusion in the wrist and femoral internal fixation removal [6]. After the surgery, he exhibited persistent fever, gastrointestinal symptoms, and a desquamating rash. Despite initial negative cultures, he ultimately tested positive for rare Gram-positive cocci from a wound swab and was treated with penicillin and clindamycin. Unfortunately, his condition deteriorated, leading to congestive heart failure, renal failure, and disseminated intravascular coagulation, resulting in his death after two cardiac arrests.

A more recent case involved a 62-year-old male who presented with confusion, fever, and hypotension a week after open reduction and internal fixation of a left wrist fracture [7]. He presented with a generalized maculopapular rash upon admission. Despite receiving fluids, vasopressor support, and appropriate antibiotics, his presentation worsened. His blood cultures eventually revealed *S. aureus*, prompting a multidisciplinary decision for surgical intervention, which included wound exploration, debridement, and removal of the metal plate. This approach led to his condition improving significantly, resulting in full recovery.

Recent case reports continue to demonstrate the diverse presentations of TSS, including atypical presentations in elderly patients following mechanical trauma and cases arising from recreational injuries such as scuba diving accidents [8,9]. These reports reinforce that TSS should be considered in any patient with rapid clinical deterioration following tissue injury, regardless of the mechanism or clinical setting. Several factors may predispose orthopedic procedures to TSS, including hematoma formation, providing a reservoir for bacterial growth, as seen in our patient with "murky fluid with hematoma" found during surgical exploration. The presence of foreign material, such as an implanted hardware, may also facilitate biofilm formation.

## Conclusions

This case highlights several critical points, including (1) the importance of interdisciplinary collaboration, (2) the recognition that TSS can present with subtle local findings while causing dramatic systemic toxicity, and (3) the value of performing a systematic diagnostic approach using the CDC’s criteria to identify and treat systemic toxin-mediated conditions. The CDC’s criteria for TSS require fever ≥38.9°C, rash with subsequent desquamation, hypotension, multisystem involvement (≥3 organ systems), and negative cultures except at the site of infection. Our patient met all these criteria with fever (101.6°F), diffuse erythematous rash, hypotension requiring fluid resuscitation, involvement of renal, hepatic, and hematologic systems, and negative blood cultures with MRSA isolated only from the surgical site. Although skin biopsy findings were non-specific, the systematic dermatologic assessment of the rash, in conjunction with clinical deterioration, was pivotal in prompting surgical re-exploration. As such, recognition of TSS should rely on clinical examination and context, with emphasis on characteristic rash and symptoms of systemic toxicity.
